# Designing a Cocreated Intervention with African American Older Adults for Hypertension Self-Management

**DOI:** 10.1155/2018/7591289

**Published:** 2018-06-03

**Authors:** Kathy D. Wright, Carolyn H. Still, Lenette M. Jones, Karen O. Moss

**Affiliations:** ^1^College of Nursing, Discovery Themes-Chronic Brain Injury, The Ohio State University, Columbus, OH 43210, USA; ^2^Frances Payne Bolton School of Nursing, Case Western Reserve University, Cleveland, OH 44106-4904, USA; ^3^School of Nursing, University of Michigan, Ann Arbor, MI 48109, USA

## Abstract

Hypertension is a lifelong disease that requires self-management. Additionally, there are disparities in hypertension self-management that disproportionately affect African Americans. Interventions designed in collaboration with older adults have the potential to improve hypertension self-management. The purpose of this design paper is to describe the process in which African American older adults and nurse researchers cocreated an intervention to address stress in the self-management of hypertension. A semistructured interview guide was used to elicit feedback on self-management behaviors to cocreate an intervention with the participants. Participants provided constant iterative feedback on the design used for the intervention. Participants prioritized the content and mode of delivery. African American older adults with hypertension (*N* = 31; 87% women) participated in two focus group sessions. The primary stressors identified by the group that influenced their blood pressure self-management were as follows: (a) measuring blood pressure and using home blood pressure monitors; (b) difficulty communicating with family and friends; (c) sleep management and pain at night; and (d) healthy eating. Based on the participants' feedback, we created four biweekly (2-hour) group sessions that incorporated their suggestions and addressed their concerns. Health care providers can use this technique to engage African American older adults in participant-centered hypertension self-management.

## 1. Introduction

Hypertension self-management is complex, particularly among older African American individuals who are affected by additional factors. These factors that are thought to contribute to disparities in hypertension include low self-efficacy, limited social support, increased stress due to racism or discrimination, and perceived lack of control over whether or not one will develop hypertension [1–3]. In addition, socioeconomic disadvantages (i.e., low income, low education, and neighborhood safety) [3, 4] increase the cumulative stressors that African Americans experience and contribute to acute and chronic stress responses that interfere with self-management and produce poor health outcomes [3, 5].

Hypertension self-management includes taking prescribed medications as directed, managing daily stress, eating a balanced diet, and performing regular physical activity, each of these is associated with improved outcomes [6]. The frequency with which these activities are performed differs by race [4], with the poorest self-management and clinical outcomes being reported in African Americans [4, 5]. Because hypertension is a lifelong disease that requires self-management, there is a need to better understand the hypertension self-management strategies employed by African American older adults to improve blood pressure control [7, 8].

Studies of hypertension self-management, however, frequently do not account for the full range of contextual factors that influence African American older adults' decisions to engage in self-management behaviors. For example, African Americans who perceive stress as the cause of their hypertension are less likely to engage in self-management behaviors [9]. In addition, interventions designed on behalf of these older adults do not take into consideration personal preferences. Increasing attention is being focused on how to design an appealing and effectively tailored program for African American older adults with hypertension. Yet, little is known about the needs and preferences of African American older adults who could benefit from hypertension self-management. This insight is critical to designing and testing patient-centered interventions that are feasible and acceptable to African American older adults.

An innovative approach to gaining critical insights into designing patient-centered interventions is cocreation.* Cocreation* is a collaborative approach to engaging stakeholders in solving complex problems. Unlike community-based participatory research, cocreation can be used with smaller groups in situations when involving an entire community may not be feasible [10, 11]. In cocreation, a stakeholder is defined as an individual that can contribute to resolution of a problem and benefit from the new solution. [11]. The stakeholders are invited to collaborate with others, asked to identify the problem, and work toward an acceptable solution. Cocreation as a methodology comes from the business management literature and has been recently used in health services research to connect those in academia with clinicians, patients, and other consumers [10, 11]. Advantages of cocreation include that it engages stakeholders early and in a “real-world setting,” which has the potential of increasing dissemination [11]. Successful cocreation includes creativity and shared governance with stakeholders in developing an intervention [10].

As an example, pharmacists used cocreation between physicians and patients to reduce inappropriate polypharmacy in the primary care setting [12].* Deprescribing*, the process of stopping medications where the risks outweigh the benefits, was the focus of the cocreated intervention. The researchers identified providers as primary stakeholders and cocreation partners. Anderson et al. [12] conducted a literature review and held focus groups with 20 general practitioners to create a deprescribing program. The general practitioners wanted to have interactive training workshops, as mechanism for identifying at-risk patients. The workshops also allowed the option of referring the patient to an expert. After integrating the literature and focus group feedback, the researchers worked with a general practitioners and a computer programmer to design a software query. The query was designed to be used with the existing electronic medical record software [12]. The intervention cocreated by the researchers and general practitioners was designed to be practical and protocol-driven, to ease the burden of use during the patient encounter. The outcomes they expected to improve were a reduction of unnecessary medications and increased patient and general practitioner satisfaction.

Based on the previous evidence supporting cocreation, we decided to cocreate a hypertension self-management intervention with community-dwelling African American older adults. The purpose of this paper is to describe the process of cocreation to develop a hypertension self-management intervention in our sample.

## 2. Materials and Methods

We used the cocreation approach to conduct focus groups to develop an intervention for hypertension self-management with African American older adults. In our study, the older adult is the stakeholder who engages in self-management of health. The focus group design was used to gather participants' perspectives on engaging in self-management activities such as sleep hygiene, exercise, diet, meditation, prayer, smartphones, healthy self-management behaviors, and other participant-generated stress reduction activities. The focus group study was approved by the University Hospitals of Cleveland Institutional Review Board.

### 2.1. Participants

A sample of community-dwelling African American adults aged 60 and older with a self-reported diagnosis of hypertension were recruited. Potential participants were assessed from the general community and using an established research participant registry maintained by the principal investigator (PI) from previous research conducted in the community. Participants were screened by phone to ensure that enrollment criteria were met. Inclusion criteria were as follows: (1) self-identified as African American, (2) a diagnosis of hypertension, and (3) a reported age of 60 years or older. Exclusion criteria were as follows: (1) non-English speaking or (2) a diagnosis of severe cognitive impairment. Based on their availability to attend the focus group sessions, participants self-selected into two groups (a Wednesday group and a Saturday group). Each of the groups met twice for session that lasted two hours.

### 2.2. Materials

A demographic data sheet was developed for age, gender, and race. A digital voice recorder and paper for writing field notes were used for the focus group sessions. A graphic recorder was present during one of the focus groups and systematically graphed (using pictures and words) the thoughts expressed by the participants. REDCap data management and survey program was used to store the data on a secure server.

### 2.3. Procedure

The focus group and the cocreated intervention sessions were held in a private meeting room at a local older adult community center. Additional details related to the convenience and comfort of participants (e.g., free parking and venue near participants' homes) were used in selecting the location for the focus groups and subsequent participant cocreated intervention. To mitigate issues related to transportation, bus passes ($5.00 each), taxi cabs (average cost $30.00 round trip), or gas cards ($5.00 each) were provided to each participant to defray the cost of travel. The participants were surveyed as to the best dates and times to meet. The schedule was created and adapted so that the maximum number of participants could attend.

At the beginning of each session, the PI made introductory comments and asked each participant to speak one at a time without the use of personal identifiers to maintain privacy. Three graduate student-nurse research assistants (two African American women and one Native American man) were present to assist with notetaking and facilitation of the discussion. Participants were reminded at the beginning of each meeting, as indicated in the consent form, that the session would be audio-recorded. Cocreated ground rules were initially established and maintained for each session. The session began with focus group questions to elicit their preferences for hypertension self-management.

To determine the types of activities participants might want and suggestions for adaptation, the PI demonstrated proposed activities. The PI demonstrated a 15-minute mindfulness exercise, a strategy to reduce stress and promote relaxation [13]. Participants then completed the mindfulness exercise while in a seated position. In addition, a single content component of a kindness-based meditation was demonstrated, delivered by using the free Stop, Breathe & Think™ application. Finally, we discussed the Dietary Approaches to Stop Hypertension (DASH) diet [14], commonly recommended to people with hypertension, and each participant was provided a copy of the diet [15]. Participants were free to opt out of any of the activities. We elicited feedback on each of the activities to develop the cocreated intervention.

Investigators collected field notes during the focus group sessions in addition to audio recordings. Debriefings were held after each focus group session among with principal investigator and research assistants to review field notes to identify the preferred content for an intervention. During the debriefing session, we listed out the common themes that arose from our field notes and direct observation. Using an iterative process and member fact checking [16], the content of the intervention was verified. In the follow-up session with participants, we used fact checking to test the components of the intervention. We used brainstorming with the participants to prioritize the list of preferred interventions.

**Figure 1:**
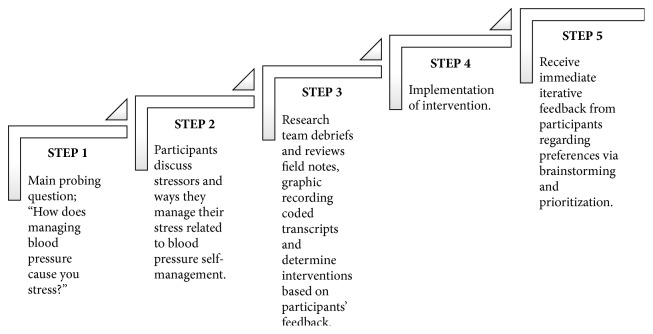
Steps to develop the cocreated intervention from focus group sessions.

Participants returned 14–21 days later for a second focus group. The PI provided a sample presentation of the cocreated intervention based on the feedback and preferences from the previous focus group sessions. Brainstorming was used to design the content of the cocreated intervention [17]. The top 3–5 recommendations from the group were included in the intervention design. A detailed report of the content analysis that led to the intervention development that included three rounds of coding is under review in [17].  Figure 1 illustrates an example of the decision process that was used to provide content for the intervention.

At the end of each focus group, participants received an honorarium in the form of a $50 gift card. Participants that participated in both groups received a total of $100. If a participant was unable to attend the second focus group session, we offered a one-on-one appointment with a research staff member, to ensure that their preferences for the intervention content would be included in the cocreated intervention.

### 2.4. Analytical Approach

Data analysis began after data were collected from the first focus group. The research team met between focus groups to begin designing a prototype of the intervention that was reviewed with participants at each focus group session (two cohorts met twice each). Data processing focused on identifying salient themes that informed the subsequent focus groups. Each transcript was checked for accuracy against the audio recordings. All transcribed data were deidentified, and audio files were destroyed once the transcripts were verified. A beginning list of themes was reviewed and verified by participants during each focus group session, which led the discussion for the cocreated intervention. This manuscript presents information on the design process used for the cocreated intervention. The results from the qualitative analyses are presented in a separate paper [17].

Investigator generated satisfaction surveys adapted from Bowen et al. [18] were distributed to gather data regarding overall satisfaction with the intervention, intent to use the intervention in the future, appropriateness of the intervention, and cultural relevance. The questions were on a visual analog scale with responses ranging from 1 (very poor) to 10 (excellent).

## 3. Results

**Figure 2:**
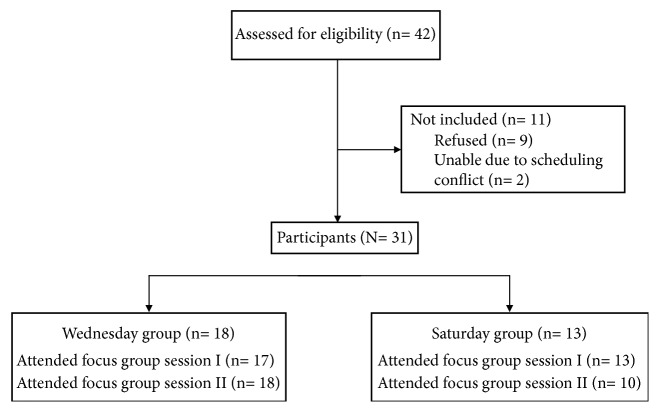
Recruitment, enrollment, and participation in focus group sessions.

Table 1 displays the demographic characteristics of the sample. Of the 49 African American older adults that were screened by phone, 31 were enrolled and 87% were women (*n* = 27).  Figure 2 lists the number of participants who were screened, enrolled, and attended the Saturday or Wednesday session. Eighteen participants were assigned to Focus Group 1 (Wednesday group) and thirteen belonged to Focus Group 2 (Saturday group). Thirty participants participated in Session I and 28 participated in Session II. The Saturday group and the Wednesday group met two times and had an average attendance of 15 participants. One participant attended both Saturday and Wednesday. One participant switched after the first Saturday session to the Wednesday group due to work conflict. 

*Mode of Delivery of the Cocreated Intervention*. The participants unanimously agreed that they wanted a group delivered intervention as opposed to an one-on-one intervention. The participants guided intervention delivery by deciding on the topics to be covered, number of sessions, type of experts they wanted to deliver the education session (e.g., requested a dietitian), how long the sessions should last (2 hours), and number of sessions that they wanted. They also selected the time of day and venue for delivery of the interventions. They told us the type of homework that they wanted to do between sessions (e.g., completing a sleep diary and food diary and logging their blood pressure at home). Participants did not want the presenter of the sessions to dominate the conversation. They wanted time for suggestions and answers. They wanted time also to provide peer support to each other in the form of recipe exchanges and cooking appliance recommendations (e.g., one person recommended using the participant compensation to purchase a vegetable steamer). 

*Prioritized Topics for the Cocreated Intervention*. The primary stressors identified by the group that influenced their blood pressure self-management were as follows: (a) measuring their blood pressure and using home blood pressure monitors; (b) difficulty communicating with family and friends; (c) organizing sleep management and pain at night; and (d) determining ways to engage in healthy eating. Based on participants' feedback, we created four biweekly (2-hour) group sessions that incorporated their suggestions and concerns. One health provider (either a registered nurse or licensed dietitian) led each session. Exercise as a way to self-manage hypertension was not brought up by participants in the focus groups.

At the request of the focus group members who helped to cocreate the intervention, the researchers successfully sought permission from the IRB to retain the participants in order for them to “try out” the cocreated intervention. An addendum to the original protocol and consent form were approved by the University Hospitals of Cleveland IRB. The series was titled “Team Learning to Take Control (TLC),” and the individual sessions were named “TLC-Monitoring Your Blood Pressure,” “TLC-Communication,” “TLC-Sleep and Pain,” and “TLC-Healthy Eating and Learning Portions (HELP).” 

*Satisfaction with the Cocreated Intervention*. In order to assess the participants' satisfaction with the cocreated intervention, we asked each participant to complete a four-item survey. Twenty-six surveys were completed and five were missing at random. Concerning satisfaction with the intervention, 100% responded with a score of eight or higher. Ninety-nine percent stated they would continue using what they learned from the intervention. The appropriateness of the intervention was rated 8–10 by (*n* = 24, 92.3%) and rated 6 by (*n* = 2, 7.7%) of the respondents. The cultural relevance of the intervention was rated 8–10 (*n* = 22, 84.6%) and rated 7 by (*n* = 4, 15.4%) of the respondents.

## 4. Discussion

This purpose of this paper was to describe the process of cocreating an intervention for hypertension self-management. Using cocreation in our study between the nurse researcher and the older adult allowed the participant (rather than the academic) to drive the design of the intervention. Participants guided intervention content by telling us what they wanted kept or removed. Participants shared their concerns around communicating with family, understanding blood pressure, and coping with the challenges of following a healthy diet. The majority of participants in the study highly rated the intervention. This may be due to their investment in the design of the intervention.

The use of a cocreated hypertension self-management intervention with a group of community-dwelling African American older adults is novel. The literature is scant on the development, use, and effectiveness of cocreated interventions. Previous studies have shown that cocreation has been effective in developing interventions [12]. The benefits of bringing together a group of individuals who share some commonalities toward a common goal are not novel [10, 11]. Using the cocreation technique in health education has the potential to have far-reaching benefits to populations at high risk of facing chronic or even co- or multimorbidities.

Although there are inconsistencies about the effects of ethnicity and gender on research participation, the group and researcher ethnic background can potentially influence participation in the focus groups [19]. Having facilitators and interventionists of a similar ethnic background may have led participants to feel more comfortable about disclosing information. We did not collect any additional data that could have led support to this assumption.

A commonly recommended intervention by nurses to patients with hypertension is exercise. However, the participants did not mention exercise as a way to self-manage hypertension. Although it was discussed in brief in the intervention delivery as appropriate, we did not push the idea of adopting an exercise routine nor make it the sole focus of an intervention session because that would have taken the power and control away from the participants.

The cocreation method is not without its challenges. One major benefit and challenge to cocreation is shared governance over the process. Interference with this process could lead to misguided results. This allowed us to demonstrate how the community-based participatory tenet of trust building over time may work in this sample of older adults with hypertension from conception of the cocreated idea to implementation. Accommodations are often needed to promote participation. For example, in a polypharmacy study, the researchers indicated that it was difficult to schedule meeting times due to the busy nature of the clinic environment [12]. This was not a challenge for our study because our participants were mostly retired or had part-time jobs. They did, however, have transportation challenges and care responsibilities for grandchildren that we took into consideration to schedule meetings. There were a couple of occasions where a participant who was a grandparent needed to bring a grandchild to a focus group meeting. As a group, the members were willing to be flexible.

While our study provides insight into working with community-dwelling African American older adults with hypertension, additional studies are needed to examine cocreated interventions in diverse samples and across chronic illnesses. Cocreated interventions allow further examination of specific self-management strategies and specific skill sets of patients. The next steps of the cocreated intervention will include (1) analyzing our data to determine what the participants found most useful and (2) using assistive technology to manage medication routines.

## 5. Limitations

Due to the positive responses to participation in the focus groups, each group was larger in size than typically recommended. This may have further reduced the likelihood that individuals who are already less likely to speak up in a group setting to do so. Additionally, the male to female ratio in the groups may have reduced the likelihood of the male perspective being included in the discussion. In a mixed gender sample, there could be less to bring up sexually related topics. As a universal limitation of focus groups, the generalizability of results is limited.

## 6. Implications for Nursing Practice

Nurses lead development of educational interventions. Cocreation helps reduce the power imbalance in research settings. Cocreation helps establish the nurse researcher and participant as equal partners [20]. The methodology used in this study to promote health education can be incorporated into around other common topics to promote health education in a nonthreatening setting with the use of peer support. Cocreation can be applied in community-based settings, such as faith-based and civic organizations. There are potential benefits to transforming this process into other homogenous groups (i.e., other disease states or age groups).

Changes will be made to the cocreated intervention based upon the feedback of the participants such as increasing the number of sessions from four to six and providing more examples of healthy ethnic recipes. Because of the age of the focus group participants in this study, there is a potential for changes made to their self-management practices to influence their social support system across the lifespan. If the participants were caring for their grandkids, they would be able to lead by example through grocery shopping practices and cooking habits. Future adaptations might include a family component and a group for men.

## 7. Conclusions

We present our experience cocreating an intervention through focus groups. Other researchers can use cocreation techniques to develop interventions. The benefits of interventions developed in this manner may be heightened by virtue of addressing what the participants indicate would most help them.

## Figures and Tables

**Table 1:** Demographic characteristics of community dwelling older adults with hypertension.

Variable participants (*N* = 31)	*n*	Percentage
Gender		
Female	27	87%
Male	4	13%
Age (years)		
60–69	13	42%
70–79	12	39%
80–89	5	16%
90–99	1	3%
Ethnicity		
Not Hispanic or Latino	23	74%
Hispanic or Latino	8	26%
Race		
White/Caucasian	0	0%
Black/African-American	31	100%

## Data Availability

The data are housed at Case Western Reserve University, Frances Payne Bolton School of Nursing. Readers interested in obtaining secure access to deidentified data will need to contact Carolyn Still, Ph.D., RN (coinvestigator), at Carolyn.Still@case.edu, Case Western Reserve University, 10900 21 Euclid Avenue, Cleveland, OH 44106-4904.
